# 3D Bioprinted Patient‐Specific Extracellular Matrix Scaffolds for Soft Tissue Defects

**DOI:** 10.1002/adhm.202200866

**Published:** 2022-09-23

**Authors:** Anne Behre, Joshua W. Tashman, Caner Dikyol, Daniel J. Shiwarski, Raphael J. Crum, Scott A. Johnson, Remya Kommeri, George S. Hussey, Stephen F. Badylak, Adam W. Feinberg

**Affiliations:** ^1^ Department of Biomedical Engineering Carnegie Mellon University Pittsburgh PA 15213 USA; ^2^ McGowan Institute for Regenerative Medicine University of Pittsburgh Pittsburgh PA 15219 USA; ^3^ Department of Materials Science & Engineering Carnegie Mellon University Pittsburgh PA 15213 USA

**Keywords:** decellularized extracellular matrix, FRESH 3D bioprinting, patient‐specific scaffolds, regenerative medicine, volumetric muscle loss

## Abstract

Soft tissue injuries such as volumetric muscle loss (VML) are often too large to heal normally on their own, resulting in scar formation and functional deficits. Decellularized extracellular matrix (dECM) scaffolds placed into these wounds have shown the ability to modulate the immune response and drive constructive healing. This provides a potential solution for functional tissue regeneration, however, these acellular dECM scaffolds are challenging to fabricate into complex geometries. 3D bioprinting is uniquely positioned to address this, being able to create patient‐specific scaffolds based on clinical 3D imaging data. Here, a process to use freeform reversible embedding of suspended hydrogels (FRESH) 3D bioprinting and computed tomography (CT) imaging to build large volume, patient‐specific dECM patches (≈12 × 8 × 2 cm) for implantation into canine VML wound models is developed. Quantitative analysis shows that these dECM patches are dimensionally accurate and conformally adapt to the surface of complex wounds. Finally, this approach is extended to a human VML injury to demonstrate the fabrication of clinically relevant dECM scaffolds with precise control over fiber alignment and micro‐architecture. Together these advancements represent a step towards an improved, clinically translatable, patient‐specific treatment for soft tissue defects from trauma, tumor resection, and other surgical procedures.

## Graphical abstract

Creation of a large, clinically relevant patient‐specific scaffold made of decellularized extracellular matrix (dECM) for large soft tissue defects, like volumetric muscle loss (VML). The scaffold is fabricated using image segmentation and freeform reversible embedding of suspended hydrogels (FRESH) 3D bioprinting of dECM bioinkand is designed to be implanted within a day of the initial injury.

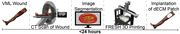

## 1 Introduction

Large‐volume soft tissue injuries to skin, fat, muscle, and other connective tissues can occur from trauma, tumor resection, and other surgical procedures.^[^ ^1^ ^]^ These injuries are often too large to heal normally on their own, resulting in scar formation, a decrease in patient quality of life due to significant functional loss, and a large financial burden on the healthcare system.^[^ ^2^, ^3^, ^4^ ^]^ For example, up to 53% of battlefield injuries involve soft tissue extremities and millions of reconstructive procedures are done annually for traumatic and oncologic resection related to muscle damage.^[^ ^5^, ^6^, ^7^ ^]^ In some cases an autologous tissue graft can be performed, but this approach is generally accompanied by donor site morbidity, loss of function, and potential graft failure.^[^ ^8^, ^9^ ^]^ Further, in the context of severe trauma associated with combat‐related injuries, a viable donor site may not be available.^[^ ^9^ ^]^ Regenerative medicine provides a potential solution to these large‐volume soft tissue defects by augmenting the body's own capacity to repair itself.^[^ ^10^ ^]^ Recent work has shown that decellularized extracellular matrix (dECM) scaffolds can be used to improve tissue regeneration, such as in volumetric muscle loss (VML), by modulating the immune response to promote constructive healing. This has been demonstrated in vivo ranging from small and large preclinical animal models all the way through early human clinical trials.^[^ ^11^, ^12^, ^13^, ^14^, ^15^, ^16^ ^]^ The dECM scaffolds serve as a physical structure that supports macrophage and progenitor cell infiltration as well as contains a reservoir of growth factors, extracellular vesicles (e.g., matrix‐bound nanovesicles), and other pro‐regenerative biomolecules that drive skeletal muscle growth, vascularization, and reinnervation.^[^ ^12^, ^14^, ^17^, ^18^ ^]^ However, while results have been promising, there have been a limited number of ways to create these dECM scaffolds, which have been limited to powders, sheets, and weak hydrogels.^[^ ^17^, ^19^, ^20^ ^]^ For complex and large injuries, none of these techniques are sufficient to completely fill the wound volume or provide an engineered environment to guide the regenerative process.

As an additive manufacturing approach, 3D bioprinting is uniquely positioned to address this challenge by using clinical imaging data such as magnetic resonance imaging (MRI) or computed tomography (CT) to create a scaffold that is anatomically accurate.^[^ ^21^ ^]^ Indeed, 3D printed plastic models from MRI and CT are now widely used for patient education, physician training, and surgical planning at hospitals worldwide, highlighting the fact that a similar 3D printing workflow is already clinically viable.^[^ ^22^ ^]^ However, bioprinting poses unique challenges in contrast to plastic because the printed material is a hydrogel bioink that may also incorporate living cells. There are a wide range of 3D bioprinting methods that have been developed, and even a wider range of bioinks, but here we focus on dECM bioinks because of the pro‐regenerative properties these have shown in animal models and human clinical trials.^[^ ^11^, ^12^, ^13^, ^14^ ^]^ As a biomaterial and scaffold, dECM has been widely used in its intact form, dried into sheets, ground into lyophilized powders, or solubilized into a hydrogel precursor that can be cast or electrospun. Yet none of these can be used to build *de novo* dECM scaffolds with controlled microstructure and overall anatomic shape. Instead, dECM, which is predominantly collagen type I along with other tissue‐specific extracellular matrix (ECM) proteins, growth factors, matrix‐bound nanovesicles, and biomolecules, can be solubilized into a solution and then solidified into a hydrogel.^[^ ^19^, ^20^, ^23^ ^]^ Recent work has shown examples of dECM bioprinting for a variety of applications such as adipose, skin, and muscle tissue regeneration.^[^ ^23^, ^24^, ^25^, ^26^, ^27^, ^28^, ^29^ ^]^ However, while dECM has been bioprinted into smaller, simplified lattice‐like geometrical shapes for VML,^[^ ^27^, ^28^, ^29^ ^]^ it has been challenging to print more complex and large 3D scaffolds required for use in large animal preclinical models and ultimately human patients.^[^ ^23^ ^]^

**Figure 1:**
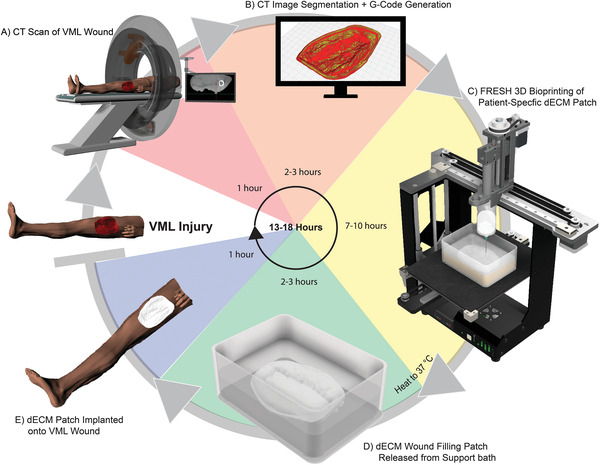
Schematic of process for creating patient‐specific dECM hydrogel patches. A) A CT scan is taken of the VML soft tissue wound and saved as a digital imaging and communication in medicine (DICOM) file. The data is imported into 3D Slicer for segmentation where a scaffold that directly matches the geometry of the wound is created. B) This CAD model is exported as an STL file and imported into Ultimaker Cura where the G‐Code of the print path is generated. C) The print is then set up using an aseptic technique, where all steps occur in a biological safety hood. Next, the patch is 3D printed using the FRESH technique. D) After the print is completed, it is placed in a cell‐culture incubator at 37 °C to melt the sacrificial gelatin support bath and nondestructively release the dECM patch. The molten gelatin is then exchanged with sterile 50 mM HEPES. E) The patch is implanted onto the wound site 12–24 h after the CT is taken. Reapplication of the patch during the healing process may be performed by repeating all the steps. Digital models of the CT scanner, human, and the MakerGear printer were obtained and adapted from GrabCad.^[^ ^66^, ^67^, ^68^ ^]^

Here, we have developed a process that combines CT or MRI imaging of a VML injury, 3D image segmentation, and embedded 3D bioprinting^[^ ^30^, ^31^ ^]^ to produce patient‐specific dECM scaffolds that can be implanted onto the wound bed (**Figure** **1**). Specifically, we used freeform reversible embedding of suspended hydrogels (FRESH) 3D bioprinting because we have previously demonstrated the ability to build organ‐scale scaffolds from MRI data using a collagen type I bioink,^[^ ^28^, ^29^ ^]^ which is the primary ECM component in the dECM.^[^ ^32^ ^]^ These acellular, dECM scaffolds do not contain cells, making them more realistic to translate than cellularized tissues because they are less expensive, easier to scale up, faster to create as no cell culture is needed, and recruit the body's own cells once implanted.^[^ ^33^, ^34^ ^]^ The FRESH process also introduces microporosity to the printed scaffolds, which has been shown to increase cellular infiltration and drive vascularization.^[^ ^31^ ^]^ Our work here has focused on a number of key advances including i) implementing an image processing pipeline to convert CT or MRI images of a volumetric tissue defect into a printable computer‐aided design (CAD) model of the patient‐specific scaffold, ii) creating a high‐concentration dECM bioink that can be used in FRESH printing with high fidelity, iii) modifying the bioprinter to use larger 25 mL syringes to produce scaffolds >35 mL in overall effective volume, iv) transferring the dECM scaffold into the wound so it makes conformal contact, v) using quantitative analysis to assess scaffold fidelity and conformality, and vi) adapting the process to a human VML injury with spatial control of the dECM density and fiber alignment. FRESH printing provides unique advantages to this approach because it enables the high‐concentration dECM hydrogel to be matched to the wound geometry and controls various aspects of the scaffold microstructure. In comparison, the high‐concentration dECM bioink is too viscous during the gelation process to be easily spread into complex wounds by hand and there is no straightforward way to control density and microstructure. Overall, this work is intended to provide a proof of concept that clinical imaging data can be used as the input to create large‐volume dECM scaffolds and covers the process from VML injury to scaffold implantation and adaptation to human VML injury anatomy. With this as a foundation, it will enable future studies to determine how the specific design of scaffold microstructure, dECM tissue source, protein composition, and mechanical properties impacts the regenerative process and functional outcomes.

## 2 Results

### 2.1 Fabrication of Patient‐Specific dECM Patches for Soft Tissue Defects

**Figure 2:**
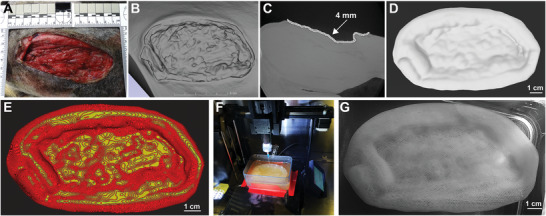
Fabrication of FRESH printed dECM patches. A) Photograph of the VML wound. B) CT scan of the wound bed. Using image segmentation, a 4 mm uniform thickness patch was created from the geometry of the wound bed. C) Side view of a slice of the CT scan of the VML wound with patch model to visualize the depth of the wound and thickness of patch. D) The patch model was cropped around the wound bed and exported as an STL. E) The CAD model of the patch was imported into Ultimaker Cura to generate the print path and create a model with 40% infill. F) FRESH 3D printed ECM hydrogel patch made of 1:1 ratio of dECM and collagen type I bioink. G) Photograph of 3D printed patch submerged in 50 mM HEPES.

We first developed our process using a canine VML model of a penetrating open wound consisting of skin, adipose, facia, and muscle tissue. A standardized size of 10 cm long, 7 cm wide, and ≈2–3 cm deep was used to create a wound in the biceps femoris of the canine (**Figure** **2**A), which is large enough to undergo substantial scarring during the healing process.^[^ ^35^ ^]^ Next, a CT scan of the wound was performed followed by volumetric segmentation to extract a solid model of the wound (Figure 2B).^[^ ^36^ ^]^ This was then hollowed to create a shell with a wall thickness of 4 mm and cleaned around the wound bed to isolate the wound patch (Video S1, Supporting Information). The 4 mm thickness was chosen because it is thick enough to produce a patch with structural integrity while still being thin enough to conform to the wound surface, however, the thickness can easily be tuned when creating the patch design. We also modified the printer extruder to hold a glass 25 mL syringe to increase the maximum bioink volume to ≈26.5 mL (Figure S1, Supporting Information). This is in comparison to commercial bioprinters that max out at a 10 mL syringe size, as does our previously published Replistruder 4 open‐source extruder design.^[^ ^37^ ^]^ The uniform thickness of the patch provides even dosage of ECM throughout the wound bed, and a side cross‐section of the patch design overlaid on the CT image of the wound confirms this (Figure 2C). After segmentation, the patch was exported as a 3D standard triangle language (STL) model (Figure 2D) and then converted into machine pathing G‐Code using slicing software (Figure 2E). An infill percentage of 40% was selected because it gave the patch sufficient structural integrity to be handleable, while still being under the maximum bioink volume that the syringe could hold. Next, the patch was FRESH 3D printed using a bioink consisting of a 1:1 mixture of 35 mg mL^−1^ purified collagen type I and 20 mg mL^−1^ of dECM from the urinary bladder matrix (UBM) (Figure 2F). This bioink mixture was selected based on in vitro bioactivity assays that assessed cytotoxicity, cell proliferation, angiogenic potential, and macrophage polarization and compared purified collagen type I to purified collagen type I mixed with dECM from UBM or skeletal muscle (Figure S2, Supporting Information). All the bioinks passed the cytotoxicity assay, however, the UBM‐collagen combination had greater cell proliferation and a greater number of macrophages with an M2 phenotype. Further, the UBM dECM has been proven to promote tissue regeneration in animal and human VML injuries,^[^ ^19^, ^20^ ^]^ while the addition of the purified collagen type I enhanced the printability of the bioink and structural integrity of the printed patches. Finally, the sacrificial gelatin support bath was melted at 37 °C to release the dECM patch, followed by washing with warm 50 mM 4‐(2‐hydroxyethyl)‐1‐piperazineethanesulfonic acid (HEPES) solution to remove residual molten gelatin (Figure 2G). The dECM patch was then ready to be implanted into the wound bed. This process was performed multiple times to establish repeatability on wounds of similar overall dimensions, but with inherent variability due to differences in anatomy and swelling post injury (**Table** **1**). Note that the overall effective volume of the dECM patch is larger than the volume of bioink used because the 40% infill leaves predefined void space within the patch.

**Table 1:** Examples of FRESH printed dECM patches for implantation into VML wounds. Six implanted dECM patches showing the overall maximum dimensions in terms of length, width, and depth. All patches were 4 mm thick with 40% infill. Overall patch volume is based on the external surface of the patch. The bioink volume accounts for the infill of the printed patch. These patches were printed at 20 mm s^−1^ and ranged from ≈5–7 h to complete

Patch	Length × Width × Depth (mm)	Overall patch volume (mL)	Bioink volume (mL)	Print time
1	118.1 × 74.5 × 19.3	37.1	23.6	5 h, 51 m
2	110.7 × 74,3 × 20.9	34.8	22.1	5 h. 23 m
3	114.4 × 73.6 × 23.3	37.2	24.6	6 h, 7 m
4	128.9 × 84.1 × 19.2	41.7	25.7	6 h, 47 m
5	118.6 × 75.3 × 19.7	38.0	24.2	6 h, 20 m
6	115.2 × 75.3 × 19.3	34.9	22.7	5 h, 40 m

### 2.2 Implantation of the dECM Patch into the Wound Bed

**Figure 3:**
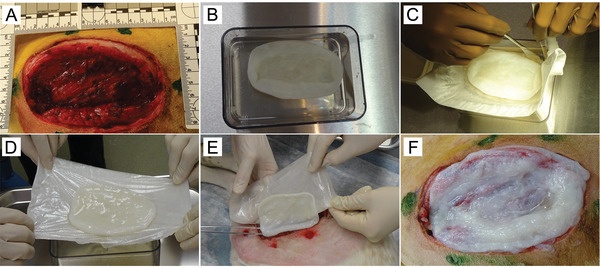
Implantation of the dECM patch onto the wound bed. A) Photograph of VML wound. B) Photograph of the FRESH printed dECM patch after thermal release from the support bath. C) Tefla‐coated gauze is carefully inserted into the solution and placed under the patch. D) Patch is slowly lifted from the HEPES solution onto the taut Tefla gauze. E) The Tefla‐coated gauze is carefully rolled under to release from the patch and sterile tools are used to assist in placing the hydrogel onto the wound bed. F)  The dECM patch in place, which conforms to the wound.

Implantation of the dECM patch into the VML wound required the development of a specific transfer process to preserve structural integrity of the patch while also enabling precise placement relative to the wound topology. Indeed, a key aspect for the clinical translation of bioprinted scaffolds is that they must be mechanically robust enough for surgical implantation.^[^ ^38^ ^]^ For the dECM patch created here, this was difficult since the patch is thin and flexible with an elastic modulus in the range of 10 kPa for the specific bioink we used.^[^ ^31^ ^]^ The first challenge we encountered was some changes in the wound shape itself due to swelling between the time of the CT of the wound and patch implantation ≈18–24 h later (**Figure** **3**A), however, as will be shown, the patch was able to adapt to these. The dECM patch, after releasing and washing, was stored in sterile 50 mMHEPES solution until use (Figure 3B). Transfer was performed using sterilized Tefla‐coated gauze, due to its non‐adherent and flexible properties, and surgical tools to help guide the patch onto the VML wound. The nonrigid Tefla‐coated gauze slightly deformed around the patch, allowing for easier manipulation of the patch onto the wound bed. To implant the patch onto the VML wound, the dECM patch was carefully maneuvered onto Tefla‐coated gauze while still immersed in 50 mM HEPES (Figure 2C). Once the patch was completely on top of the gauze, it was slowly lifted out of the HEPES (Figure 3D) and brought over the wound bed. The patch was properly oriented to the wound bed and then the gauze was rolled backward, releasing the patch onto the wound bed (Figure 2E). The repeatability of this process was demonstrated by performing it multiple times on wounds with slightly varying sizes and geometries (Figure S3, Supporting Information) and corresponding patient‐specific dECM patches (Table 1).

### 2.3 Dimensional Accuracy and Conformality of dECM Patches

**Figure 4:**
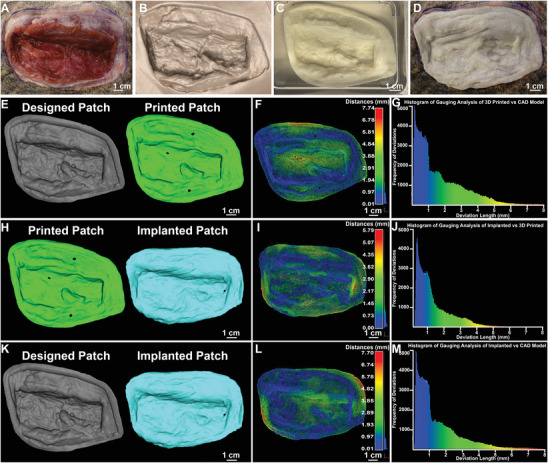
Dimensional analysis comparing the designed dECM patch model, to the FRESH printed dECM patch pre‐ and post‐implantation. A) Photograph of VML wound. B) CT scan of VML wound rendered in 3D. C) Photograph of dECM patch submerged in 50 mM HEPES solution. D) Photograph of implanted dECM patch on the wound bed. E) Original CAD model used for printing (gray) compared to the CAD model created from the CT scan of the printed patch (green). F) The color map results from gauging analysis of the printed patch compared to the original CAD model. The dark blue color indicates <0.01 mm difference, demonstrating an almost perfect fit. G) The histogram results from the gauging analysis of the printed patch compared to the original CAD model. H) The geometry of the 3D printed patch (green) was compared to the geometry of the patch post‐implantation (blue). I) Color map results from gauging analysis of the implanted patch compared to the as printed patch. J) Histogram results from gauging analysis of the implanted patch compared to the asprinted patch. K) The geometry of the original cad model (gray) was compared to the geometry of the patch post‐implantation (blue). L) Color map results from gauging analysis of the implanted patch compared to the original CAD model. M) Histogram results from gauging analysis of the implanted patch compared to the CAD mode used for printing.

The dECM patch is intended to match the topology of the VML wound and to be conformal so that the dECM is in direct contact with the tissue. To assess this, a VML wound was created post‐necropsy in a canine limb (**Figure** **4**A), imaged by CT (Figure 4B), and then the limb was frozen to maintain structural integrity. These studies were performed postmortem because it facilitated the CT imaging of the VML wound pre‐ and post‐implantation of the dECM patch. We incorporated 2% of barium sulfate into the bioink as an X‐ray contrast agent to aid visualization.^[^ ^31^ ^]^ Also, because there was a freeze‐thaw cycle in‐between the pre‐ and post‐implantation CT images, this produced some minor shape changes that helped to mimic swelling post‐injury. To determine dimensional accuracy, three different aspects of the dECM patch were compared throughout the process. First, a CAD model of the dECM patch was created to match the VML wound, as previously described. The dECM patch was then FRESH 3D bioprinted and CT imaged before (Figure 4C) and after implantation onto the wound (Figure 4D). The CT images were used to create CAD models of the 3D printed patch in each state using image segmentation. Gauging analysis was then performed to compare the original 3D CAD model to the printed patch before and after implantation onto the wound bed to assess i) print accuracy and ii) the adaptability of the dECM patch to fit the wound geometry post‐injury. The FRESH printed dECM patch had an average deviation of ±1.55 mm compared to the original CAD model (Figure 4E–G), confirming that we can print the patch with high fidelity with variations of 1%–2% relative to the total length and width. Comparing the dECM patch pre‐ and post‐implantation showed an average deviation of ±1.14 mm (Figure 4H–J), demonstrating that the implantation process deformed the patch by 1.5% or less relative to the total length and width. Finally, comparing the implanted dECM patch to the original CAD model showed an average deviation of ±1.38 mm (Figure 4K–M), establishing the similarity between the initial CAD design and the final implanted patch morphology. While this analysis was performed for a single patch, it establishes the ability to FRESH 3D print the dECM patch with high fidelity that is also maintained after implantation. A closer look at the heat maps (Figure 4F,I,L) shows that the regions of greatest deviation are associated with the periphery of the patch and some of the more centrally located crevices in the wound. Mismatch at the periphery is to be expected since some skin contracture around the wound is known to occur in the actual VML injury as well as during the freeze‐thaw cycle performed in this specific analysis.

**Figure 5:**
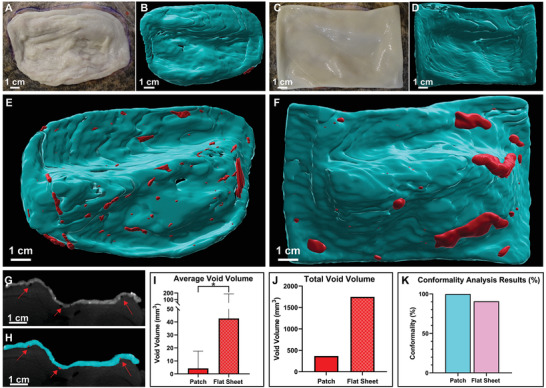
Conformality Analysis and Void Space Quantification of Implanted Patch and Flat Sheet. A) Photograph of implanted dECM patient‐specific patch. B) CAD model of patch created in Imaris to visualize the surface. C) Photograph of implanted dECM flat sheet. D) CAD model of flat sheet created in Imaris. E) CAD model of backside of patient‐specific patch that interfaces with the wound bed where the void spaces can be visualized in red. F) CAD model of backside of the flat sheet that interfaces with the wound bed where the void spaces can be visualized in red. G‐H) A visualization of a (G) side profile of the wound bed with implanted patch volume and (H) an ortho‐slicer profile of the exact position showing the patch conformality in that slice. Void spaces are indicated by the red arrows. I) Quantification of the average void volume, data presented as mean ± SD, *n* = 87 for patch voids and *n* = 45 for flat sheet voids. *P*‐values are calculated from an unpaired t‐test using log‐transformed data, *P < 0.05. J) Quantification of the total void volume for the implanted patch and flat sheet. K) Conformality analysis results which compare the surface area of the top of the wound bed to its respective dECM prints. All models were created in 3D slicer and the prints were cropped directly around the wound bed to ensure that the surface area was only being quantified for the extent of the wound geometry. CAD models were then imported into MeshMixer to determine surface area.

Conformality of the dECM patch to the surface of the wound bed is also important because the dECM needs to be in contact with the tissue. To establish that the patient‐specific geometry is required to achieve conformal coverage, a flat sheet large enough to cover the entire wound was printed out of the dECM bioink as a control. Both the dECM patient‐specific patch (**Figure** **5**A,B) and the dECM flat sheet (Figure 5C,D) were implanted onto the same frozen wound, imaged by CT, and then image processed to asses conformality through contact area and void volume. After further image segmentation, the contours of the dECM patient‐specific patch were highlighted in blue and the voids between the patch and the wound were highlighted in red (Figure 5E). This process was then repeated for the dECM flat sheet (Figure 5F). A cross‐section of the dECM patient‐specific patch on the wound shows that there were black regions at the interface between the two surfaces that were air pockets (Figure 5G). The image segmentation was performed to isolate the dECM patient‐specific patch and quantify the size of these voids (Figure 5H). There were 87 voids detected on the dECM patient‐specific patch, with the majority located in places where there were deep contours. Although there were only 41 voids detected on the implanted dECM flat sheet, these voids were significantly larger compared to the patient‐specific patch (Figure 5I). This discrepancy in void volumes between the patient‐specific patch and flat sheet can be further visualized in Videos S2 and S3, Supporting Information. The total void volume associated with the flat sheet was ≈4.8‐fold larger than that of the patient‐specific patch (Figure 5J). The conformality of the print to the wound bed was also evaluated based on the surface area of each that were in contact, and the flat sheet regions that extended beyond the perimeter of the wound were cropped to fit the outline of the wound (Figure S4, Supporting Information). The surface area of the dECM patient‐specific patch touching the wound was 99.9%, showing an excellent fit to the wound geometry (Figure 5K). Achieving this degree of conformality does require good implantation technique, as air bubbles can be inadvertently trapped between the patch and the wound. The implantation technique we developed (Figure 3) does minimize this and helps properly position the dECM patch relative to the wound topology. In contrast, the surface area of the dECM flat sheet touching the wound was 90.3%, demonstrating a poorer fit to the wound geometry. The void analysis shows that this poorer fit was due to multiple areas with large gaps between the wound and the flat sheet. Together, these data demonstrate that we can successfully print a conformal dECM patient‐specific patch and that the deformability can adapt to small variations in wound geometry and match most of the contours. It also demonstrates that the patient‐specific geometry does create a more conformal fit with less void space between the print and the wound bed compared to a nonspecific dECM flat sheet.

### 2.4 Fabrication of a Patient‐Specific dECM Patch for VML in a Human Quadricep

**Figure 6:**
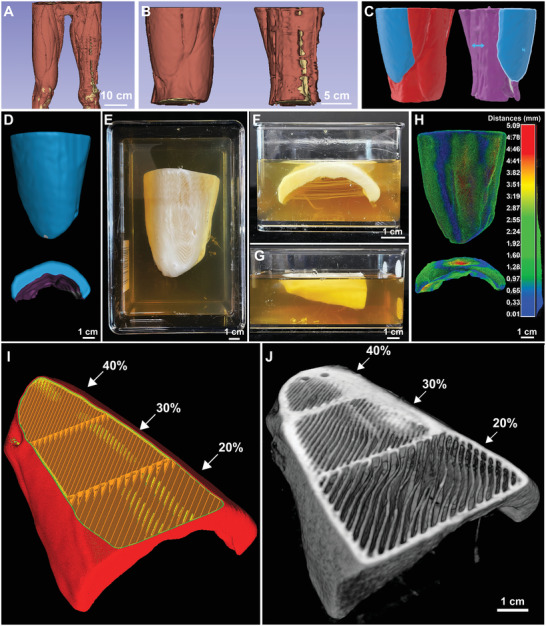
Patient‐specific dECM scaffold with spatial control for implantation into a human VML injury. A) Image segmentation was utilized to create the model, and B) the model was cropped to a smaller section above the knee. C) The scaffold was created by isolating the vastus lateralis muscle from the uninjured leg and overlaying it onto the injured top of the isolated injured leg model‐aligning the bone and outer section. D) A “subtraction” Boolean modifier was then used to create a model from the difference between the isolated healthy muscle and the injured leg. The model was then imported into MeshMixer to cut out the center section and smooth the rough edges. This model was then sliced to generate the G‐Code and printed with 1:1 ratio of collagen type 1: UBM with 2% barium sulfate. E‐G) Photographs of the printed scaffolds from top and side views. H) Dimensional gauging analysis of the 3D printed scaffold to the original CAD model. **I)** The infill angle was modified to be 45° to match the innate muscle fiber alignment of the vastus lateralis muscle, and infill density was varied from 20%–40%. J) 3D visualization from a CT scan of structure with an exposed slice to show the varying infill percentages and 45° fiber alignment to match the muscle alignment of the vastus lateralis muscle.

As a further step, we adapted the process developed for the canine VML injury and applied it to a clinically relevant human VML injury to demonstrate the ability to use the CT data to design a patient‐specific dECM scaffold based on the un‐injured contralateral leg. Deidentified CT scans of a closed VML injury in a human patient were selected because the contralateral leg was uninjured and could be used to determine the volume of muscle needed to be regenerated. To start, the CT data was used to generate a 3D model of the injured and uninjured legs (**Figure** **6**A). The models were then cropped to isolate a section where the VML injury occurred in the left leg, demonstrating a partial compartment loss above the knee (Figure 6B).^[^ ^39^ ^]^ Further segmentation of the uninjured leg was used to isolate an intact and full volume of the vastus lateralis muscle, a muscle that underwent significant injury on the contralateral side. This was then used as the reference model to fabricate the dECM scaffold (Figure S5, Supporting Information). To do this the vastus lateralis muscle was isolated in the uninjured leg, mirrored to fit the orientation of the injured leg, and then overlayed onto the injured leg to ensure the alignment matched that of the uninjured leg (Figure 6C). The final geometry of the scaffold was the difference between the uninjured and injured muscle (Figure 6D). The scaffold was then scaled to 70% of its original volume with dimensions of 8.6 × 6.7 × 3.5 cm, which was done in order to reduce the amount of bioink that would be required to print it due to the syringe size limitation of 25 mL. This scaffold was FRESH printed with the collagen type I and dECM bioink with barium sulfate as a contrast agent (Figure 6E–G) and imaged by CT. Gauging analysis of the external surface comparing the original CAD model to the FRESH printed scaffold showed good dimensional accuracy with an average deviation of ±1.47 mm (Figure 6H), which was comparable to average deviations found for the dECM patches used for the canine VML experiments (Figure 4).

Finally, it may be beneficial to regionally control infill alignment and density of the dECM bioink to enhance regeneration. As a proof of concept that we can modify distinct parts of the scaffold, we varied the infill density to increase in the region where the muscle attaches to the tendon. For this, infill density was varied in different sections of the scaffold, at either 20%, 30%, or 40%. The infill pattern can also introduce topographical cues within a scaffold, which have been shown to enhance cellular alignment.^[^ ^40^ ^]^ The infill pattern was modified to create a unipennate (oriented at one fiber angle) structure, at 45 degrees, to match the orientation of the myofibers of the vastus lateralis muscle (Figure 6I), and to potentially enhance cellular alignment along muscle fiber orientation. The structure and infill alignment were visualized in 3D (Figure 6J), clearly demonstrating the ability of the FRESH printing approach to control the arrangement and density of the dECM material.

## 3 Discussion

Here, we have fabricated patient‐specific patches made from a dECM bioink and have demonstrated the constructs’ fidelity, dimensional accuracy, and conformality using a canine VML model. To do this required several important achievements that have advanced the biofabrication field. First, we created a high‐concentration bioink that consisted of 10 mg mL^−1^ dECM from UBM and 17.5 mg mL^−1^ purified collagen type I. Since some of the main components of the decellularized UBM include collagen type I, III, and VII,^[^ ^41^, ^42^ ^]^ we estimate that the total collagen concentration is >20 mg mL^−1^. This work builds directly off our previous publications on FRESH 3D bioprinting collagen,^[^ ^31^, ^43^ ^]^ extending it to dECM. While previous work has used dECM bioinks, they have been unable to recapitulate large, complex architectures.^[^ ^23^ ^]^ Second, we have printed constructs using a large volume of bioink, which required us to modify our open‐source Replistruder 4 extruder^[^ ^37^ ^]^ to use a 25 mL Hamilton Gas Tight syringe. While we previously built a FRESH bioprinter to use a 60 mL plastic syringe to print a full‐size alginate human heart scaffold, this approach used a more compliant syringe material, which resulted in reduced control compared to our new design.^[^ ^44^ ^]^ Since this work was a proof of concept, we limited the total volume of our scaffolds to a single syringe, but larger scaffolds can be fabricated by simply using multiple extruders. Third, we demonstrated excellent fidelity, dimensional accuracy, and conformality using quantitative gauging from CT images. The printed dECM constructs ranged from 2–12 cm in length, width, and height, but only had an average deviation of ≈1.5 mm. Some of this deformation was intentional as we wanted the dECM patches to be able to conform to small changes in wound geometry due to swelling. Fourth, we developed an image analysis, FRESH bioprinting, and surgical implantation process that could go from a CT scan to patch implantation in 12–18 h. The single longest step was the FRESH bioprinting, which ranged from 5–7 h (Table 1), however, this is an area that can readily be accelerated by using faster printers and/or multiple extruders at the same time. Of course, as with any extrusion‐based printing approach, print time is directly related to printing parameters such as print speed, nozzle diameter, infill percentage, and scaffold complexity. Therefore, while the dECM patches presented here are scaled to a canine size, there is nothing from a technical standpoint preventing us from printing the 2–4 times larger scaffolds necessary for treating VML in human patients.

The objective of this paper was to lay the groundwork for FRESH printing large, patient‐specific scaffolds made from dECM to improve tissue regeneration. We used VML as a soft tissue defect model because VML wounds vary in geometry, size, and location,^[^ ^45^ ^]^ demonstrating the ability of FRESH‐printed scaffolds to adapt to different injuries. For optimal regeneration, the macro‐architecture of the engineered construct should be designed around each patient's specific injury.^[^ ^8^ ^]^ However, the micro‐architecture is another important scaffold property that can influence the regeneration process. We selected a rectilinear pattern at 40% infill because this is a standard lattice structure used in 3D printing. This produced dECM filaments that were ≈400 µm in diameter which were spaced ≈1000 µm apart. This is effective for providing mechanical strength, as demonstrated by the ability to handle and implant the dECM patches (Figure 3). However, this is essentially an isotropic micro‐architecture and not designed to direct the structure of the regenerating tissue. Instead, it is possible to have the micro‐architecture specifically designed with the intent to control how cells infiltrate and organize during regeneration. There is an extensive body of literature describing how microscale fibers and patterns can direct the anisotropic formation of aligned muscle tissue.^[^ ^46^, ^47^, ^48^, ^49^, ^50^, ^51^, ^52^ ^]^ Here we demonstrated that these types of fibers can be FRESH printed within a large dECM scaffold (Figure 6). Specifically, the scaffold fiber orientation and density can be spatially controlled, influencing the number of alignment cues and mechanical properties (Figure 6I,J). In the future, we could print multiple bioinks into a single dECM patch to spatially control mechanical properties and composition,^[^ ^53^ ^]^ as we have previously shown that collagen‐based bioinks can be printed in the same FRESH support bath along with ionic, enzymatic, and photo‐cross‐linkable bioinks.^[^ ^31^ ^]^ Specific growth factors shown to improve muscle regeneration, such as hepatocyte growth factor (HGF) and insulin‐like growth factor 1 (IGF1), could be incorporated into one or multiple bioinks to further guide and enhance muscle regeneration.^[^ ^10^, ^53^ ^]^ With FRESH we can combine these approaches to bioprint a scaffold where alignment cues, growth factors, ECM composition, mechanical properties, and other characteristics can be varied spatially to enhance and direct muscle and soft tissue regeneration and thus improve integration with the surrounding, uninjured tissue.

There are of course some potential limitations of the approach, specifically the use of acellular versus cellularized scaffolds. Some of the research on VML repair has demonstrated that the addition of cells onto a dECM scaffold improves functional recovery in rodent VML injury models.^[^ ^54^, ^55^ ^]^ However, we intentionally focused on an acellular approach because the addition of cells complicates the clinical translation of the scaffolds for a few reasons. First, unless autologous cells are seeded on the scaffold, the patient will need to be put on immunosuppressants that have a long list of side effects including kidney and liver toxicity. Second, using autologous cells requires a biopsy followed by in vitro expansion to get sufficient cell numbers for seeding, a process that is expensive, time‐consuming, and prevents application in an emergency setting. Our approach has already demonstrated that the scaffold can be implanted onto the wound bed within 24 h of the initial CT scan of the injury, and this can be accelerated further. Third, the addition of cells changes the medical device classification from a biological to a combination device, which will prolong the FDA approval of the device and significantly increase development costs.^[^ ^56^ ^]^ Finally, the acellular dECM scaffolds have already demonstrated safety and efficacy in humans in initial clinical trials, providing clinical evidence of VML repair.

The methods and results presented here are a first step towards the long‐term goal of soft tissue regeneration using 3D bioprinted dECM scaffolds. A large body of work has already demonstrated the potential of dECM to serve as an instructive scaffold to modulate the immune response towards constructive healing.^[^ ^57^, ^58^, ^59^, ^60^ ^]^ This includes our previous work showing that dECM sourced from porcine UBM can regenerate VML in vivo in mouse models and human patients.^[^ ^11^, ^14^ ^]^ However, while these published studies have highlighted the approach's potential and helped decipher the underlying mechanisms of repair, the sheets, powders, and weak hydrogels used have not been able to fully restore tissue volume in most applications. This is where 3D bioprinting provides unique capabilities, because the design of patches and scaffolds can be based directly on clinical imaging data from CT and MRI. This is an entirely digital workflow from imaging, to computer‐aided design, to bioprinting, then followed by surgical implantation. Patient‐specific 3D printed constructs, such as surgical guides, thermoplastic skull plates, and orthopedic implants, are now commonplace and provide improved performance for the patient through better anatomical fit.^[^ ^61^ ^]^ FRESH 3D bioprinted dECM scaffolds should provide similar benefits by enabling the delivery of specific amounts of dECM to different areas of the injury, while also controlling micro‐architecture and mechanical properties that can influence regeneration. The work presented here is focused on the methodology and results associated with fabrication and validation, a critical step given the size and complexity of the printed scaffolds. Future studies will focus on how the dECM composition and micro‐architecture of the patches and scaffolds impacts muscle regeneration in vivo, as well as how reapplication of the dECM patch during the healing process can further improve functional outcomes for open VML wounds.

## 4 Conclusions

Here we have demonstrated patient‐specific, dECM patches that can be bioprinted using the FRESH technique. Patient specificity has long been a key aspect of the argument for 3D printing in healthcare.^[^ ^62^, ^63^ ^]^ By utilizing CT imaging, image segmentation, and the FRESH printing process, we generated large scaffolds that directly match the geometry of large soft tissue defects. Moreover, these scaffolds were robust enough for implantation onto the wound, and conformal to the surgically generated wounds. We also demonstrated the fabrication of a scaffold for a human VML model, which modified infill architecture and alignment throughout the scaffold. While large constructs have been bioprinted before,^[^ ^44^, ^64^ ^]^ our dECM patches represent the largest bioprinted ECM scaffolds yet reported. They are also the first bioprinted dECM scaffolds to be successfully tailored to an individual patient's wound with clinically relevant sizes.

## 5 Experimental Section

### Surgical Procedure

Approval was obtained from the University of Pittsburgh Institutional Animal Care and Use Committee (protocol 191 050 701). Six female canines (*Canis familiaris)* with weights of 17–21 kg were subjected to a muscle defect consisting of a unilateral resection of the biceps femoris muscle. Prior to sedation, the right lateral thigh of each canine was shaved in preparation for surgery. Each canine was anesthetized by intravenous administration of either 2% thiopental sodium or 6–10 mg kg^−1^ propofol followed by tracheal intubation. Inhalant isoflurane (1%–5%) was used to maintain a surgical plane of anesthesia throughout the surgery. The surgical site was prepared with an aseptic wash of 70% ethyl alcohol followed by a topical 10% povidone‐iodine solution. After aseptic preparation, the greater trochanter and lateral epicondyle of the femur were identified to establish anatomic landmarks for entry. A 10 cm long by 4 cm wide uppercase “I” shape was marked to establish the initial incision point halfway between the greater trochanter and lateral epicondyle. Skin was incised along the outline of the “I” shape and the skin was retracted for visualization of the biceps femoris. A 10 cm × 4 cm template was used to mark the outside border of the planned defect. Scalpel dissection was used to minimize unnecessary cauterization and potential burn damage to the tissue. In the instance of hemorrhage, bleeding was controlled with electrocautery. The ≈2 cm defect was removed, and the overhanging skin was removed leaving an open window to the wound. The canines received buprenorphine (0.02 mg kg^−1^) postoperatively and the leg was dressed in Tefla‐coated nonstick gauze and Vet Wrap bandage. The bandage was replaced every 1–3 days for up to 10 days. All canines received postoperative antibiotic prophylaxis for the first 5 days (cephalexin 25 mg kg^−1^) and an analgesic every 12 hours for the first 5 days (buprenorphine 0.02 mg kg^−1^). Each animal was observed daily for routine clinical parameters including temperature, appetite, activity, and ability to bear weight on the operated leg. Animals were fed a high‐energy, high‐protein diet (advanced protocol high‐density Canine diet; PMI Nutrition LLC, Henderson, CO) with unlimited access to water.

### Computed Tomography

CT scanning of the wound was performed immediately post‐operation, anesthesia was induced with propofol (6–10 mg Ml^−1^, IV) and maintained with inhaled isoflurane (1%–2%) during scans. CT scans were performed using a Vimago GT30 (Epica Animal Health, San Clemente, CA) with proprietary High‐Definition Volumetric Imaging (HDVI) technology. Animals were placed on the scanning bed in the lateral position with the wound facing upward and the stifle joint positioned at 90° flexion. Approximately, a 175 × 175 × 300 mm field of view centered on the wound was obtained with the following settings: 90 kV, 35 mA, 7–8 ms, and a voxel size of 0.35 mm × 0.35 mm × 0.35 mm. All CT scans were stored as digital imaging and communication in medicine (DICOM) files.

### Generation of a Wound‐Filling dECM Hydrogel Patch Model from CT Imaging

After the CT scan was downloaded as a sequence of images, the data series was imported as DICOM files into 3D Slicer (versions 4.10 and 4.11, https://www.slicer.org). Using the “3D segmentation editor”, a new segmentation was created from the data series. In the segmentation editor, the “automatic thresholding” tool was utilized to extract the VMLwound into a 3D model. The exact threshold values were manually chosen based on selected slices in XY, XZ, and YZ planes. The wound bed segmentation was then inverted using the “logical operator” tool to create a volume that was directly conformal to the surface of the wound bed. A 4 mm uniform‐thickness shell was then created from the external surface of the inverted bed using the “hollow” tool, generating the wound‐filling ECM hydrogel model. Subsequently, the ECM hydrogel model was manually cleaned around the wound bed to remove obvious errors and excess volume using the “cut” and “erase” tools. The fringes of the model were then trimmed to decrease the total volume of the wound The photographic images of the wound provided by the surgical team were used as a guide. After trimming, the whole ECM hydrogel patch was smoothed using the “smooth” tool and a “median filter” with 3‐pixel window, to decrease unnecessarily detailed features. When the model was complete, it was exported as an STL file for G‐code generation and printing.

### G‐Code Generation

The wound‐filling dECM hydrogel patch was imported into Ultimaker Cura (Version 4.3.0, https://www.ultimaker.com/software) for slicing. For the data presented here, a 23‐gauge needle (Jensen Global) was used with a 25 mL Hamilton gastight syringe (Hamilton Company, Series 1000). The main print settings were 0.16 mm layer height, 20 mm s^−1^ print speed, 40% infill, 2 perimeters, 2 top, and bottom layers, and 100% flow tweak. Rectilinear infill was used with connected infill lines (50% infill overlap) and retraction and combing were both turned on. The ECM hydrogel model was oriented to allow it to fit into the print container (13.5″ × 9.5″ × 3″) and with the wound‐facing side oriented upwards and the long axis of the patch parallel to the *x*‐axis.

### Sterile FRESH Gelatin Microparticle Support Bath

The FRESH gelatin microparticle support bath was generated via a complex coacervation method to produce gelatin microparticles using protocols previously described.^[^ ^31^ ^]^ Briefly, a powder mixture of 3.0% (w/v) gelatin Type B (Fisher Chemical), 0.125% (w/v) Pluronic F‐127 (Sigma‐Aldrich) and 0.3% (w/v) gum Arabic (Sigma‐Aldrich) was ultraviolet (UV) treated for at least 20 min. Then, powders were dissolved in a 50% (v/v) 200 proof ethanol in DI water solution at 45 °C in a sealable 4 L Nalgene polypropylene container and the pH was adjusted to ≈5.65 by addition of 1 M hydrochloric acid. An overhead stirrer (IKA, Model RW20) with the metal rotor passed through a sealed bearing, was then used to maintain mixing. All steps previously described took place in a biosafety hood. The container was then sealed to minimize evaporation and maintain sterility as the mixture was moved to a temperature‐controlled room (at 21 °C) and cooled to room temperature while stirring overnight at 550 RPM.

To remove the ethanol and Pluronic F‐127 from the gelatin microparticles, the solution underwent a series of washing steps performed in a biosafety cabinet. The rotor was turned off at least two hours prior to washing to allow the microparticles to gravitationally separate from the aqueous supernatant. The container was placed back into a biosafety hood. The liquid supernatant was decanted using 50 mL serological pipettes. The resulting solution was transferred into 6–8 sterilized 250 mL polycarbonate Nalgene containers using a serological pipette and then centrifuged at 450 g for 3–5 min. The supernatant was aspirated using a serological pipettor and the gelatin microparticles were resuspended in sterile distilled water (dH_2_O). The support bath was then washed three times with dH_2_O at 750 g, 750 g, and 1000 g for 3–5 min each, aspirating the supernatant and replacing it with fresh dH_2_O between each wash. For the final washing step, sterile 50 mM HEPES solution (Corning), at pH 7.4, was added to the Nalgene containers. They were then centrifuged at 1000 g for 5 min. The supernatant was aspirated, fresh sterile 50 mM HEPES was added, and sterile support/HEPES solution was pipetted into a sterile container and stored at 4 °C. Prior to printing, the uncompacted support bath solution was pipetted into sterilized 100 mL syringes and degassed in a sterilized vacuum chamber for 15 min, followed by centrifugation at 2000 g for 5 min. The supernatant was removed, and the gelatin microparticle support bath was transferred into a sterilized print container. For all aseptic processes, all the plastic reusable materials were sterilized using ethylene oxide (EtO), all metal was sterilized using dry heat (250 °C for at least 30 min), and for dH_2_O, the water was sterilized using dry heat then filtered through a 100‐micron Nalgene rapid‐flow filter. All other liquid solutions used sterile dH_2_O and were filtered through the Nalgene rapid‐flow filter.

### Bioactivity Analysis of Bioink

Three different bioink formulations consisting of purified collagen type I (COL1), decellularized urinary bladder matrix (UBM) blended with COL1 (UBM‐COL), and decellularized skeletal muscle matrix blended with COL1 (SKM‐COL) were evaluated. These bioinks were FRESH printed into square‐shaped hydrogel scaffolds 0.8 cm^2^ (*n* = 4 per bioink composition) and used for all bioactivity studies.

For the cytotoxicity assay, scaffolds were incubated in Dulbecco's high glucose modified Eagles medium (DMEM, Cytiva HyClone) at 1 cm^2^ mL^−1^ extraction ratio for 24 h at 37 °C temperature under 300 RPM agitation. The extract was further diluted to a 1:2 and 1:4 ratio with DMEM media containing 10% fetal bovine serum (FBS) (Atlanta Biologicals, S11150) and 1% antibiotic (Gibco 15 240 062) to get 50% and 25% extracts, respectively. Next, 3T3 fibroblasts (ATCC) were incubated with the scaffold extracts for 24 h at 37 °C and 5% CO_2_. The viability of cells was determined using an MTT cytotoxicity assay (Molecular Probes, M6494) as per the standard ISO 10993–5 protocol.

For the cell adhesion and proliferation assay, scaffolds were washed with PBS and C2C12 skeletal myoblasts (ATCC) in 20 µL DMEM media containing 10% FBS and 1% antibiotic were seeded at a concentration of 1 × 10^4^ cells per construct and incubated for 3 h. Constructs were then gently washed with DMEM media and cultured for 10 days at 37 °C and 5% CO_2_. Cell proliferation was quantified using a cell counting kit‐8 (CCK‐8, ApexBio, K1018) as per the manufacturer's protocol. Briefly, 10 µL of CCK‐8 reagent was added to the wells on days 4, 7, and 10 and incubated for 4 h. The constructs were transferred to a fresh well plate before adding CCK‐8 reagent to avoid interference with the cells attached to the plate. The supernatant media was collected and the constructs were gently washed with fresh DMEM media, followed by incubation untill the next time point. The absorbance of collected supernatant media was measured at 460 nm and compared with the standard curve plotted with different cell numbers. For fluorescent imaging of adhered cells, constructs were washed with PBS twice and fixed in 2% paraformaldehyde (methanol free, Thermo Scientific, AA433689M) for 10 min. Then permeabilized with 0.1% Triton X 100 (Sigma‐Aldrich) for 5 min and incubated in 1% BSA (Bioworld, 220 700 212) for 20 min. The constructs were washed with PBS twice and stained with 5 µL rhodamine‐phalloidin (Invitrogen, R415) in 200 µL media for 20 min. Constructs were further washed with PBS and nuclei were counterstained with 4′,6‐diamidino‐2‐phenylindole (DAPI) (Sigma‐Aldrich, D95464). The images were taken with TRITC and DAPI filter in an inverted fluorescence microscope (Carl Zeiss, Axio Observer).

For the macrophage polarization assay, murine bone marrow‐derived macrophages were isolated from 6–8‐week‐old C57bl/6 mice (Jackson Laboratories) as previously described.^[^ ^65^ ^]^ Briefly, the ends of the tibia were transected and the marrow flushed with DMEM with 10% FBS, 10% L929 supernatant, 0.1% beta‐mercaptoethanol (Sigma‐Aldrich, M3148), 1% Antibiotic‐Antimycotic, 10 mM nonessential amino acids (Cytiva HyClone, SH30238.01), and 10 mM HEPES buffer. The macrophages were plated at 1 × 10^6^ cells mL^−1^ and allowed to mature for 7 days at 37 °C and 5% CO_2_. Macrophages were activated towards an M1 phenotype for 6 h in 20 ng mL^−1^ interferon *γ* (IFN*γ* mouse recombinant protein, eBioscience, 34‐8311‐82) and 100 ng mL^−1^ lipopolysaccharide (LPS from Escherichia coli O111:B4, Sigma‐Aldrich), Macrophages were activated towards an M2 phenotype in 20 ng mL^−1^ Interleukin‐4 (IL‐4 recombinant mouse protein, Invitrogen, PMC0045). The macrophages were treated with the scaffold extracts diluted at 1:10 ratio with macrophage maturation media for 24 h. The macrophages were immunofluorescently stained with mouse primary antibodies for arginase (Abcam, ab91279) or inducible nitric oxide synthase (iNOS) (Invitrogen PA3030A) overnight at 4 °C. The macrophages were then stained with Alexa Fluor 488 anti‐rabbit secondary antibody (Invitrogen, A21206) for 1 h, followed by DAPI for 5 min. The macrophages were imaged using an inverted fluorescent microscope and the percentage of positively stained cells for each condition were determined.

For the angiogenesis assay, human umbilical vein endothelial cells (HUVECs) (ATCC) were cultured using endothelial complete growth media (Promocell GmbH, C22111). First, 1 × 10^4^ HUVECs suspended in 50 µL of media were seeded on the 3D printed constructs and cultured for 6 h in endothelial complete growth media. The cells were then stained with Calcein AM dye (Invitrogen, C3099) for 10 min to label live cells and fluorescent images were obtained. In addition, HUVECs were seeded on a layer of matrigel (Corning, 354 234) and as a positive control treated with endothelial complete media, or as a negative control treated with suramin angiogenic inhibitor (30 µM) in endothelial basal media. The cells were cultured for 6 h, and phase contrast images were obtained under 10x magnification. The formation of endothelial tubes was quantified using Image J analysis software.

### Col1‐dECM Bioink Preparation

All collagen type 1 (Col1) was purchased as LifeInk 240, a pre‐acidified collagen at 35 mg mL^−1^ (LifeInk240, Advanced Biomatrix). The urinary bladder matrix (UBM) was provided by the Badylak lab at 20 mg mL^−1^ concentrations in an acidified state. A 1:1 mixture of the Col1 and UBM was created by thoroughly mixing equal volumes of each component in two 10 mL BD syringes using a luer‐lock syringe coupler. Approximately 4 mL of UBM and 4 mL of Col1 were mixed at a time and repeated until required bioink volume was created. This whole process was performed in a biosafety cabinet to maintain sterility. After mixing, the bioink was placed overnight in a temperature‐controlled room at 21 °C to bring the ink to room temperature before removing the air bubbles. By allowing the bioink to go from 4 to 21 °C, it exposed more air bubbles to the surface that were condensed within the cold bioink. The next day, the resulting Col1‐ECM ink was centrifuged at 3000 g for 10 min to remove bubbles. If needed, the inks were spun down for an additional to 5 min at 3000 g to remove excess bubbles. For printing, the ink was transferred to an ethylene oxide (EtO) sterilized 25 mL gastight syringe (Gastight Syringe, Hamilton Company).

### Sterile Printing of Wound‐Filling dECM Hydrogel Patch

The entire printing process was performed using sterile reagents and within a biosafety cabinet to ensure aseptic technique. The bioprinter, based on a modified Makergear M2 Model E, was placed into the biosafety hood, sprayed and wiped with ethanol, allowed to air dry, then exposed to sterilizing UV light for 20 min. The 25 mL Hamilton gastight syringe containing the bioink was mounted to the Replistruder 4^[^ ^37^ ^]^ and an EtO sterilized dispense needle (23 G, Jensen Global) was mounted to the luer‐lock of the syringe. The EtO sterilized print dish was filled with support bath (≈400 mL of compacted support) via100 mL syringes (Wilburn Medical). The gelatin support bath was transferred into the print container via a EtO sterilized 100 mL syringe and a plunger. For each print, 8–10 of the 100 mL syringes were filled to the 85 mL line with sterile support. The syringes were then placed into a vacuum chamber (Bel‐Art Products), for at least 15 min to degas air bubbles. The vacuum was sterilized using ethanol spray and then UV light exposure for at least 20 min in a biosafety hood with the top of the vacuum removed and facing upwards. After the support was degassed, it was covered with sterile parafilm and sealed with sterile adhesive bandages. To compact the support, four 100 mL syringes were spun down at a time at 2000 g for 5 min. Custom centrifuge adapters were designed, and 3D printed using fused‐deposition modeling (FDM) plastic printing. After centrifugation, the supernatant was aspirated using a 50 mL serological pipette. Sterile stainless‐steel wires were then inserted into the syringes ensuring the wire touches the support. A sterile plunger was taken from an unopened bag containing a 100 mL syringe. The plunger was inserted into the syringe containing the support and pushed down until the bottom of the plunger was flush to the support. The wire was then removed. Custom‐made flanges were then tightened onto the outside syringe to help with support extrusion into the container. A 12‐gauge blunt tip needle (Jensen Global) was screwed into to the bottom luer‐lock end of the syringe. The plunger was then pushed down, extruding the support into the container in a continuous fashion. This process was repeated for each following syringe. After each separate syringe was added, the print container was covered and tapped to the bottom of the biosafety cabinet to make sure all support in the container was evenly distributed before adding support from additional syringes.

The print dish was then externally affixed to the bed of the printer using double‐sided tape and laboratory tape, with the long axis oriented along the *x*‐axis of the printer. The center of the dish was located using the dispense needle and the needle was then submerged to an appropriate Z‐depth within the gelatin microparticle support. The G‐code file for the dECM hydrogel patch was then executed by the printer using a Duet‐2 Wi‐Fi interface. Each patch took between 5–7 h to print and used ≈23–27 mL of ink, depending on variations in print size and geometry.

After the dECM hydrogel patch print was completed, the needle was retracted from the gelatin microparticle support bath. The print dish was removed from the bed of the printer and was covered with the container lid. The covered dish and sterile 50 mM HEPES were then transferred to a cell culture incubator at 37 °C for 3 h to thermally release the gelatin microparticles. After 3 h, the print dish and warm sterile HEPES were returned to the biosafety hood. Half of the molten gelatin was removed and replaced with 50 mM HEPES. This process was repeated three times. Finally, the print dish was re‐covered with its lid, sealed with parafilm, and transferred to 4 °C to gel for at least one hour. The gelatin‐embedded ECM hydrogel patch was then ready for transportation to the site at which it would be extracted and implanted into the animal.

The dECM flat sheet (117 × 90 × 4 mm) was designed in SolidWorks and printed with the dECM bioink with 2% BaSO_4_ and implanted onto the wound using the methods described below.

### Implantation of dECM Hydrogel Patch

The gelatin‐embedded dECM hydrogel patch was transported to the site where it would be implanted. The sealed print dish was placed into an incubator at 37 °C along with sterile 50 mM HEPES. When the entire bulk of gelatin was melted (≈2 h), 50% of the gelatin was removed and replaced with 50 mM HEPES. This process was repeated until the gelatin was sufficiently diluted that it did not hinder the motion of the floating dECM hydrogel patch. The container was then transferred to the surgical suite. The animal was prepared for implantation by removing previous bandaging and by administering an appropriate anesthetic. The ECM hydrogel patch was then lifted from the print container using a Tefla‐coated gauze and transported to the wound site. The gauze was brought directly over the wound bed and then slowly rolled under, releasing the patch onto the wound. The dECM hydrogel patch was inspected and manually manipulated to ensure alignment. The wound was then protected with more Tefla‐coated gauze and appropriately bandaged and covered to prevent the animal from further exposing it.

### Dimensional Gauging Analysis of 3D Printed Patch Pre‐ and Post‐Implantation

Following initiation of deep anesthesia via propofol (6‐10 mg/kg, IV) a pentobarbital (87 mg/kg, IV) overdose was used to induce death with vital signs monitored accordingly. A VML wound was created on the uninjured leg of a canine subject post‐necropsy, immediately imaged under the CT scanner, and then frozen. A patch model of the wound was created through image segmentation in 3D slicer using methods described above. Patches were printed with the dECM bioink with 2% barium sulfate (BaSO_4_) (Sigma‐Aldrich), as a contrast agent. After printing, the patch was released in an incubator at 37 °C and half of the volume of molten gelatin was exchanged with 50 mM HEPES. The print was then placed at 4 °C overnight. The next day, the print was transferred to the imaging facility and placed back in a 37 °C incubator. Once the gelatin was melted, all gelatin was exchanged for 50 mM HEPES. The print was then imaged on the CT scanner before and after implantation onto the canine leg. Scans were exported as DICOM files and imported into 3D Slicer for image segmentation. CAD models of the patch were made using the “threshold” tool in 3D Slicer, to highlight only the patch, then manually cleaned up using the “scissor tool” and smoothed using “median filter”. After the models were created, they were exported as STL files and imported into CloudCompare (version 2.11.3, https://www.cloudcompare.org/) for dimensional gauging analysis. The two models being compared were aligned using the “match bounding box centers” and “fine alignment tools” on their cloud surfaces. The “cloud‐to‐cloud distances” tool was used to detect the size of deviations between the two models. A colormap was created to visualize these deviations. The colormap‐histograms were also exported from CloudCompare. The cloud‐to‐cloud distances were then exported as American Standard Code for Information Interchange (ANSCII) files and imported into GraphPad Prism 9 (GraphPad Software) where histograms, with a bin width of 0.1 mm, where created to analyze the data. The STL models were also imported into MeshMixer (version 3.5.474, Autodesk) to quantify their volume.

### Conformality and Void Volume Analysis

For visualization of the CT image, the DICOM files were loaded into Imaris image analysis software (Bitplane, 9.5.1). Rendered 3D views, plane cuts, and histogram levels were selected to visualize the external and internal geometry of the prints. Models of the implanted patient‐specific patch and flat sheet were created by manually drawing a contoured surface in the *XZ*‐Plane to highlight each slice of the patch. The “Ortho Slicer” tool was used to help visualize the conformality of the patch to the wound surface slice‐by‐slice. Spaces between the wound and the patch (the “voids”) were visualized by using the “Baseline Subtraction”, “Background Subtraction”, and “Invert” tools in the Image Processing channel. Surfaces of the “voids” between the wound bed and the patch were created by automatic thresholding. The volume was then inspected slice‐by‐slice in the *XY*‐ and *YZ*‐planes to manually locate any missed voids. Quantitative data about the void surfaces were provided in Imaris. Conformality analysis was evaluated by comparing the surface area of the backside of the printed scaffold to the top of its respective wound. 3D models of the scaffolds and the wound beds were created in 3D slicer using “automatic thresholding” then manually cleaning up the models using the “paint” and “level tracing” tools. The models of the implanted prints (patch and flat sheet) were generated in the same file as their respective wound models. The prints were aligned on top of the wound and then cropped to ensure that each print had the same exact outer bounds as the wound it was implanted on. The CAD files were then exported as STL files and imported into MeshMixer to quantify the surface areas. In MeshMixer, the “Unwrap Brush” was used to highlight only one side of the model. The back of the prints which interfaced with the wound were highlighted, while the topside of the wounds were highlighted. The “separate” tool was used to separate the two sides of the model, and then the surface area was quantified using the “stability” tool. Each surface area quantification was performed three times each per model and averaged to account for user error since each side was manually highlighted. The percentage conformality of the print to the wound bed was determined as (Surface Area of Bottom of Scaffold)/(Surface Area of Top of Wound)*100.

### Generation of dECM Hydrogel Scaffold for Human VML Injury

The deidentified CT scans of a VML injury from a human patient was provided through the University of Pittsburgh. Since the CT scan was deidentified, no approval was required for using the human material. The DICOM files were imported into 3D Slicer for image segmentation. A CAD model of the healthy, uninjured leg, as well as the injured leg, were created by the “thresholding” tool and then manually segmenting the muscle and bone using the “paint” and “erase” tools. The vastus lateralis (outer thigh muscle) in the uninjured leg was isolated. The models were cropped above the knee and exported as STL files. The models were then imported into 3D Builder (version 18.0.1931.0, Microsoft Corporation) where the outer thigh was mirrored, and then placed on top of the injured leg. A Boolean subtraction modifier was then used to isolate the difference in the geometry. The model was then imported into MeshMixer (version 3.5.474, Autodesk) where it was cleaned up, smoothed, and exported as an STL. This model was then imported into Ultimaker Cura (version 9.10.1), where modifiers were added to the model so that the infill percentage was 20%, 30%, and 40% for each third of the model. The infill pattern was also modified to only make lines of 45°, matching the unipennate muscle fiber alignment in the native muscle. This model was then printed on a custom‐modified Aerotech AGS 1000 3D bioprinter system, at 50 mm s^−1^, using dECM with 2% barium sulfate. The next day the scaffold was imaged using the CT Scanner. The DICOM files were imported into Imaris (for 3D visualization of infill) and into 3D slicer (for CAD creation). The dimensional analysis of the CAD model of the printed patch was compared to the original CAD used for printing, using the protocol stated above. The color mapped histogram was exported from Cloud Compare.

### Statistical Analysis

Statistical analysis was carried out using GraphPad Prism 9. All data sets for the bioactivity assays were analyzed using the Kruskal‐Wallis nonparametric test with a post‐hoc analysis using Dunn's multiple comparisons test. For the cell proliferation assay, day 10 data was compared between the COL1 (*n* = 8), UBM‐COL (*n* = 8), and SKM‐COL (*n* = 8). For the macrophage polarization, two separate tests were run to either compare Arginase or iNOS expressing cells for the M0 (*n* = 12 for arginase and *n* = 7 for iNOS), M1 (*n* = 11 for arginase and *n* = 12 for iNOS), M2 (*n* = 12 for arginase and *n* = 12 for iNOS), COL1 (*n* = 12 for arginase and *n* = 12 for iNOS), UBM‐COL1 (*n* = 12 for arginase and *n* = 12 for iNOS), and SKM‐COL1 (*n* = 12 for arginase and n = 12 for iNOS) groups. The angiogenesis assays that quantified the number of nodes (positive control (*n* = 7), negative control (*n* = 6), UBM‐COL (*n* = 8), and SKM‐COL (*n* = 5)), number of tubes (positive control (*n* = 7), negative control (*n* = 6), UBM‐COL (*n* = 8), and SKM‐COL (*n* = 8)) and length of detected tubes ((positive control (*n* = 7), negative control (*n* = 6), UBM‐COL (*n* = 8), and SKM‐COL (*n* = 5)) were all calculated using separate Kruskal‐Wallis non‐parametric tests. In all cases, significance was defined as *p* < 0.05. The void volume data sets for the patient‐specific patch (*n* = 87) and flat sheet (*n* = 45) were transformed logarithmically to create a normal distribution. Any voids that were smaller than 0.001 mm^3^ were disregarded before analysis. Normality was confirmed using the Shapiro‐Wilk test (*alpha = 0.05*). Equal variances were confirmed using an *F*‐test (*P* < 0.05). A two‐tailed, unpaired *t*‐test was run on the data sets. In all cases, significance was defined as *p* < 0.05.

## Conflict of Interest

A.W.F. is the chief technology officer and an equity holder in FluidForm Inc., which is a startup company commercializing FRESH 3D printing and has license rights from Carnegie Mellon University. FRESH 3D printing is the subject of patent protection including U.S. Patent No. 10,150,258 and provisional patent No. 63/082621. S.F.B. is the chief scientific officer and equity holder in ECM Therapeutics Inc., which has license rights to MBV technology from the University of Pittsburgh.

## Author Contributions

A.B. and J.W.T.contributed equally to this work. All authors conceived the experiments and contributed to the scientific planning and discussions. J.W.T and D.J.S performed bioprinter conversions and adaptions. G.S.H performed dECM bioink generation. A.B. and R.K. conducted experiments and analyzed data for the bioactivity assays. R.J.C, S.A.J, and S.F.B performed experimental planning and surgical procedure for the canine VML model. A.B., J.W.T, and C.D. performed image segmentation and bioprinting. A.B. and D.J.S performed image analysis. A.B., J.W.T, C.D, and D.J.S. prepared final figures and text. A.B., J.W.T, C.D., D.J.S., and A.W.F. drafted the manuscript and interpreted the data.

## Supporting information

Supporting Information

Supplemental Video 1

Supplemental Video 2

Supplemental Video 3

## Data Availability

The data that support the findings of this study are available from the corresponding author upon reasonable request.
