# Supplementary material for: 3D Bioprinted Patient‐Specific Extracellular Matrix Scaffolds for Soft Tissue Defects
Source: Adv Healthc Mater. 2022 Sep 23;11(24):2200866. doi: 10.1002/adhm.202200866 (PMC9780169; doi:10.1002/adhm.202200866)
Supplement: Supplementary file 1 — Supporting Information [file ADHM-11-2200866-s001.pdf]

ADVANCED HEALTHCARE MATERIALS

Supporting Information

for *Adv. Healthcare Mater.*, DOI 10.1002/adhm.202200866

3D Bioprinted Patient-Specific Extracellular Matrix Scaffolds for Soft Tissue Defects

*Anne Behre, Joshua W. Tashman, Caner Dikyol, Daniel J. Shiwarski, Raphael J. Crum, Scott A. Johnson, Remya Kommeri, George S. Hussey, Stephen F. Badylak and Adam W. Feinberg**

Title

3D Bioprinted Patient-Specific Extracellular Matrix Scaffolds for Soft Tissue Defects

Authors

Anne Behre[1*], Joshua W. Tashman[1*], Caner Dikyol[1], Daniel J. Shiwarski[1], Raphael Crum[2], Scott A. Johnson[2], Remya Kommeri[2], George Hussey[2], Stephen Badylak[2], Adam W. Feinberg[1, 2, 3]

[1]Department of Biomedical Engineering, Carnegie Mellon University, Pittsburgh, PA, 15213

[2]McGowan Institute for Regenerative Medicine, University of Pittsburgh, Pittsburgh, PA 15219, USA

[3]Department of Materials Science & Engineering, Carnegie Mellon University, Pittsburgh, PA, 15213

*These authors contributed equally to this work and are both first authors.

Supplemental Videos

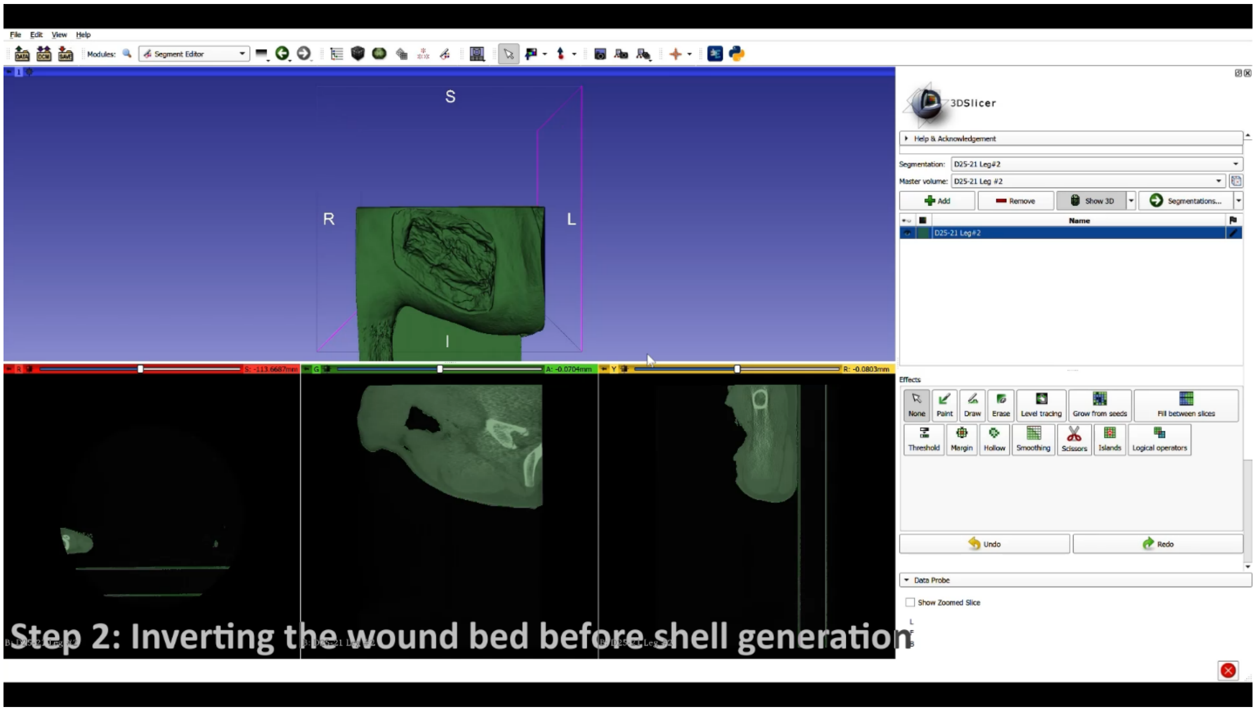

Supplemental Video 1. Image Segmentation Process to Create Patient-Specific Wound Filling Patch. The data is imported into 3D Slicer as a DICOM file. Once imported, a new segmentation is created in “segmentation editor” window. The “threshold” tool is then used to highlight the wound bed. The “logical operator” tool is used to invert the wound bed, and the “hollow” tool is used to create a 4 mm uniform thickness shell which directly matches the geometry of the wound. The “scissor” and “smooth” tools are used to cut the CAD model around the wound bed and decrease unnecessarily detailed features. The finished model is then exported as an STL and imported into CURA for G-code generation.

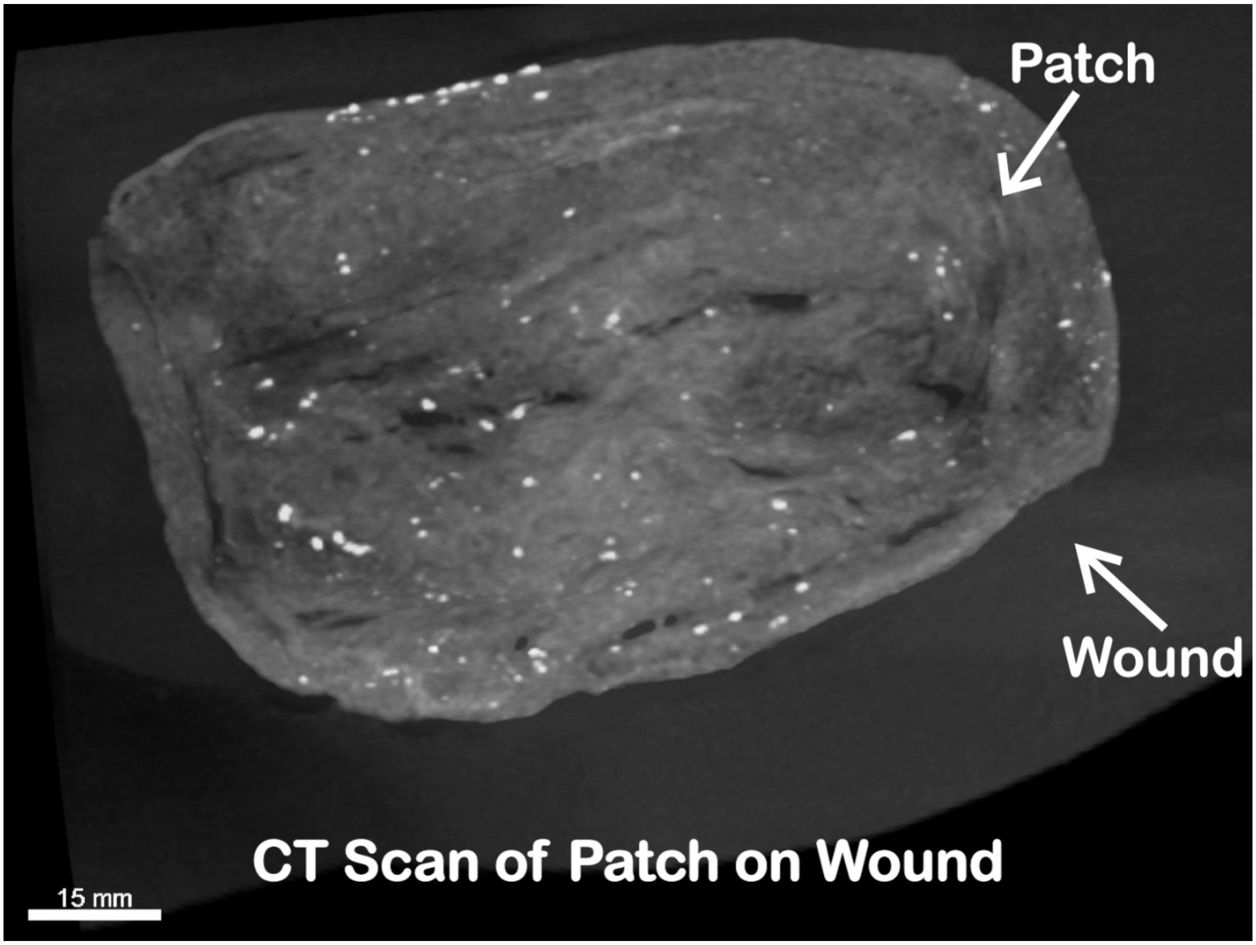

Supplemental Video 2. CT Scan and 3D Renders of Patient-Specific dECM Patch and Voids. Video of the Imaris renders which visualizes the fit of the patch to the wound bed by showing the CT scan slices of the patch on wound and the full 3D renders of the patch and voids, and renders slice-by-slice on the wound bed.

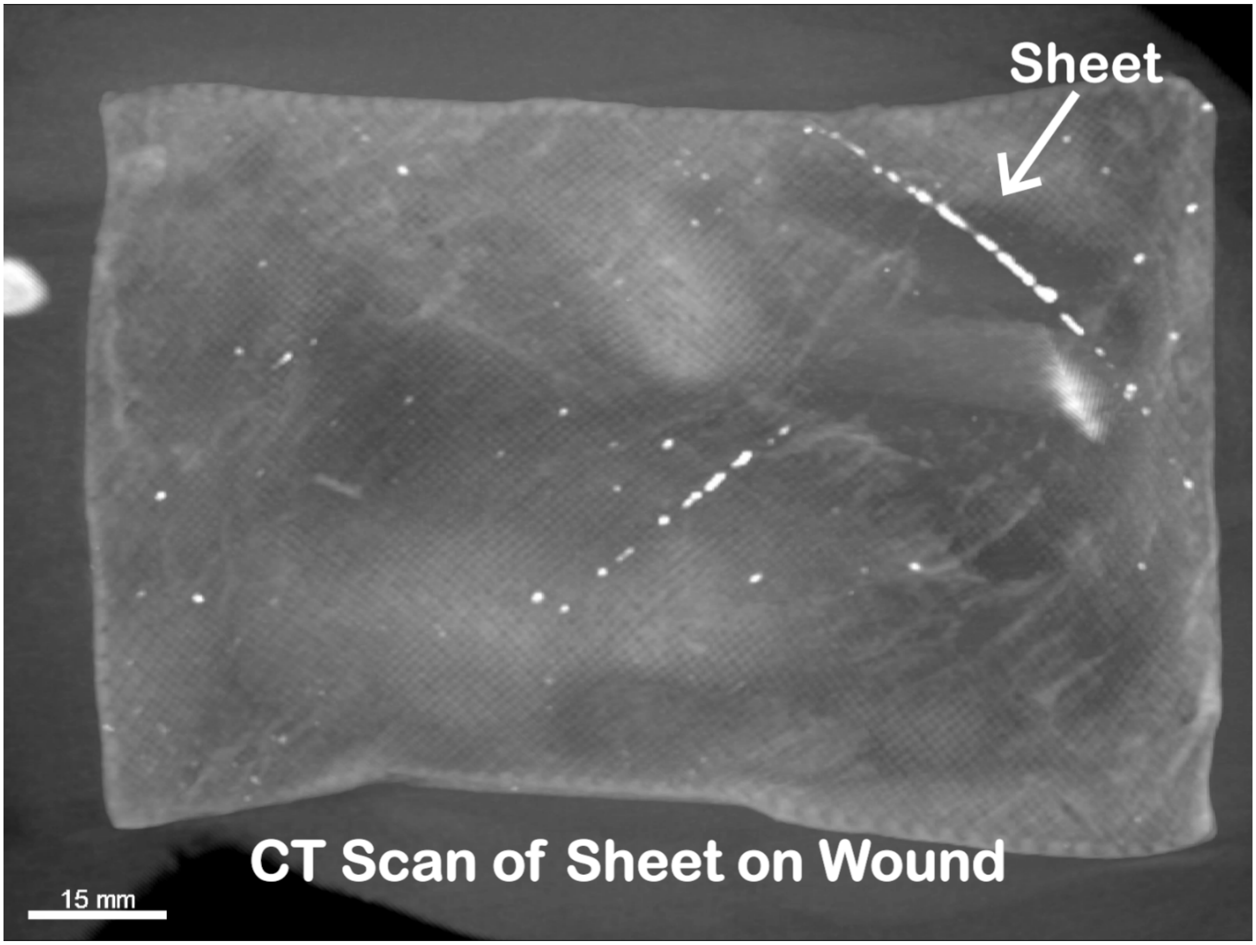

Supplemental Video 3. CT Scan and 3D Renders of dECM Flat Sheet and Voids. Video of the Imaris renders which visualizes the fit of the printed dECM flat sheet to the wound bed by showing the CT scan slices of the flat sheet on wound, the full 3D renders of the flat sheet and voids, and renders of the sheet and voids slice-by-slice on the wound bed.

Supplemental Figures

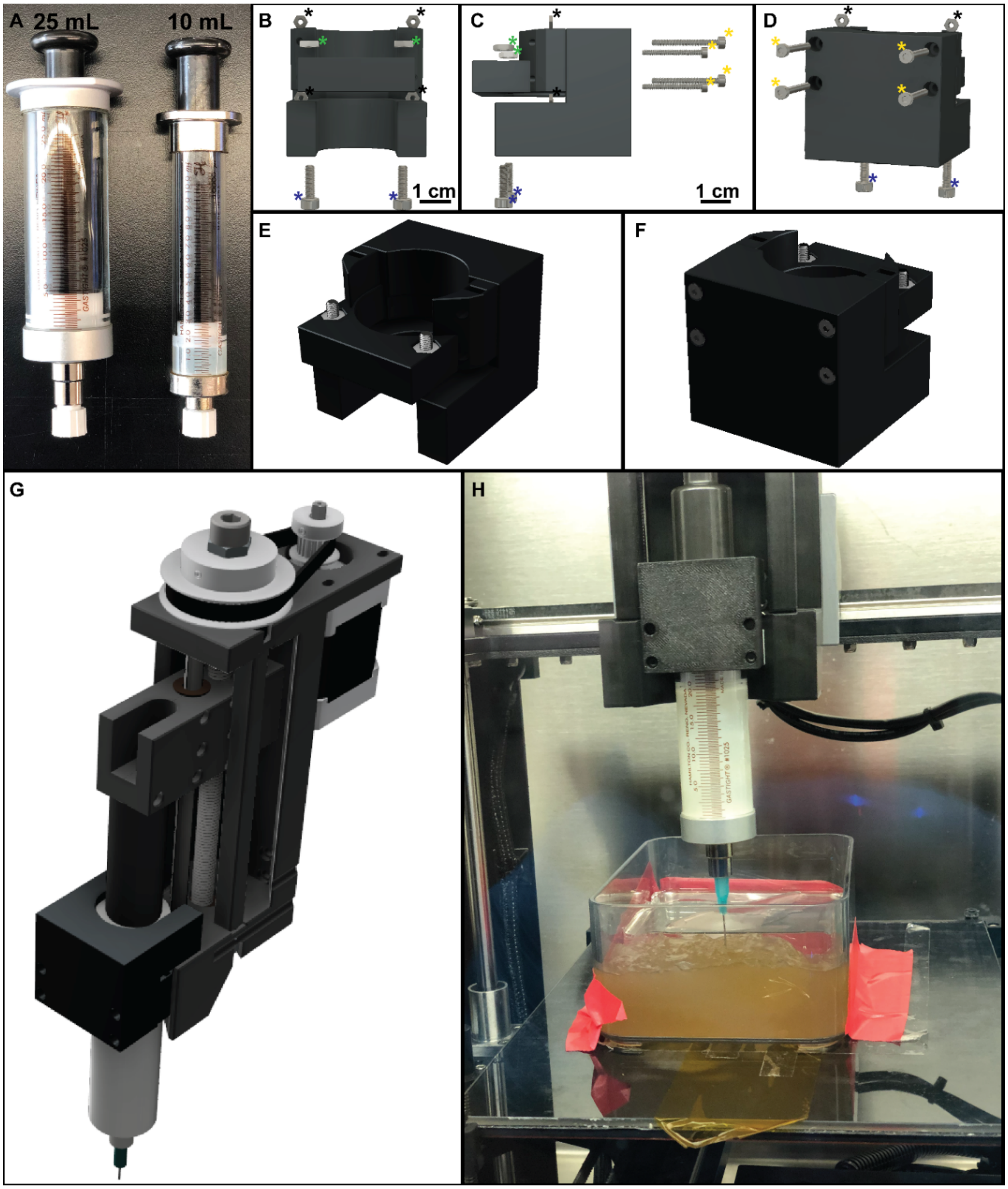

Supplemental Figure S1. 25 mL Hamilton Syringe Adapter for Replistruder 4. A 25 mL adapter for the Replistruder 4 needed to be designed since the largest syringe previously used was a 10 mL syringe. **(A)** A photograph of a 25 mL and a 10 mL syringe to visualize difference in size. **(B-D)** Exploded views of the 25 mL adapter assembly of the front **(B)**, side **(C)**, and isometric view **(D)**. A **black** asterisk indicates an M2 thin steel hex nut (part #90695A025 - McMaster-Carr). A **green** asterisk indicates an M3 thin steel hex nut (part #90695A033 - McMaster-Carr). A **blue** asterisk indicates a M3 x 0.5 mm alloy steel socket head screw (part #91290A115-McMaster-Carr). A **yellow** asterisk indicates an M2 x 0.4 mm alloy steel socket head screw (part #91290A049 - McMaster-Carr). **(E-F)** Isometric views of the 3D render of the 25 mL adapter assembly for the front that attaches to the extruded **(E)** and the back **(F)**. **(G)** Isometric view of the 3D render of the 25 mL adapter attached to the Replistruder 4. **(H)** Photograph of printing with the 25 mL Hamilton Syringe and the plastic printed syringe adapter.

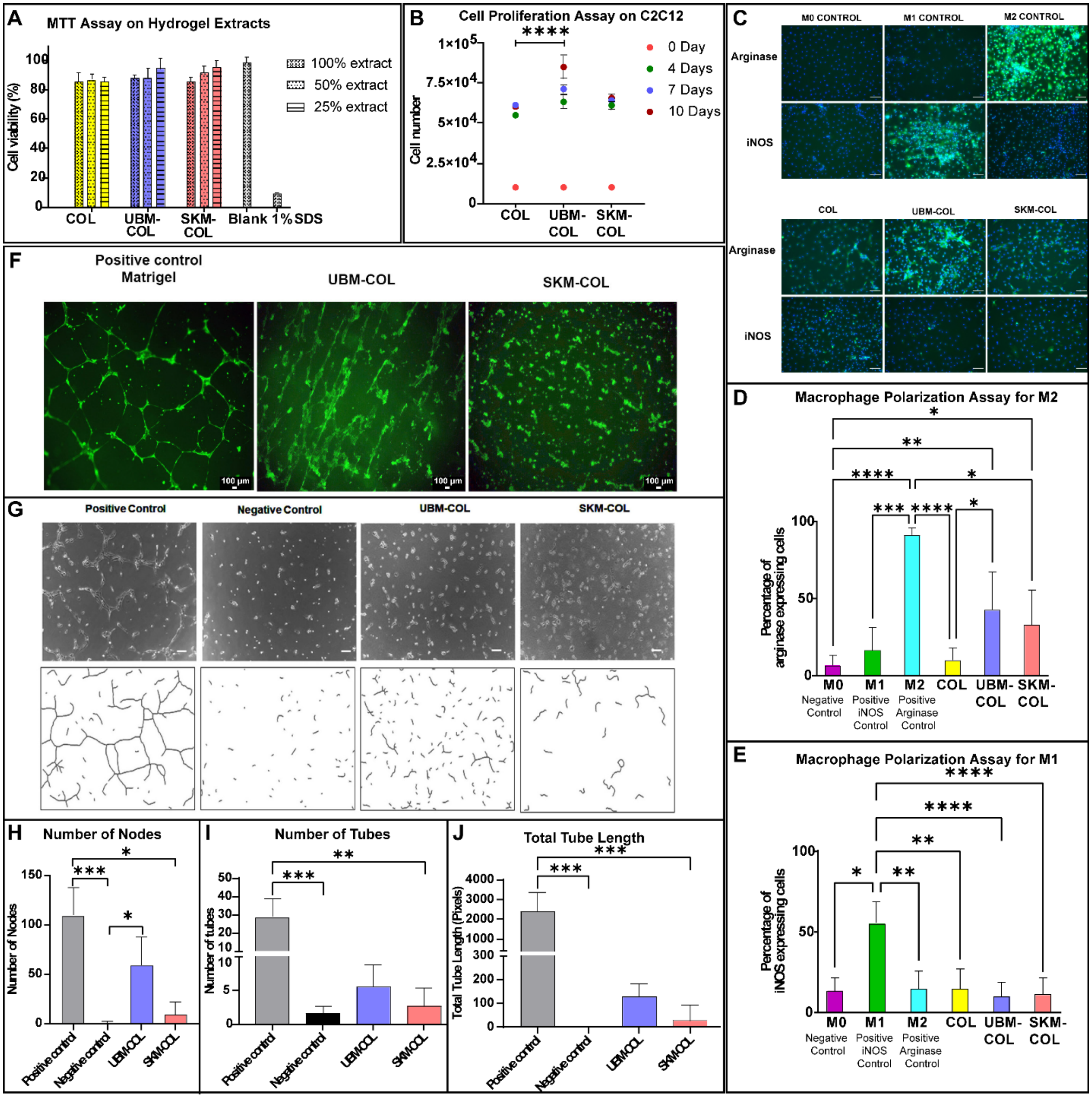

Supplemental Figure S2. Bioactivity Assay Results for Determining Bioink Composition. A. MTT cytotoxicity assay results, where all hydrogel bioinks have >80% cell viability (n=4 per bioink sample and n=3 per extract per sample).. **B.** Results from the cell proliferation assay using C2C12 cells, data presented as mean ± SD for each time point, n = 8 per time point. P-values are calculated using Kruskal-Wallis non-parametric test with post-hoc analysis of Dunn's multiple comparisons test for the 10-day timepoint, ****P<.0.0001. **C.** Fluorescent images (20X objective) of murine macrophages treated with hydrogel extract and stained with Dapi (blue) and Arginase 1 (M2) or iNOS (M1) antibody (green). Scale bars are 100 µm. **D.** Results of the macrophage polarization assay for M2 phenotype marker, data presented as mean ± SD, n = 12. P-values are calculated using Kruskal-Wallis non-parametric test with post-hoc analysis of Dunn's multiple comparisons test, *P<0.05, **P<0.005, ***P<0.001, ****P<0.0001. **E.** Results of the macrophage polarization assay for M1 phenotype marker, data presented as mean ± SD, n = 12. P-values are calculated using Kruskal-Wallis non-parametric test with post-hoc analysis of Dunn's

multiple comparisons test, **P*<0.05, ***P*<0.005, *****P*<0.0001. **F.** Fluorescent Images (5X objective) of HUVEC cells seeded on printed hydrogels and stained with Calcein AM. Matrigel is the positive control. **G.** Phase contrast images (10X objective) of HUVEC cells treated with hydrogel extract **(top)** and Image J analysis vascular tree diagrams of HUVEC cells treated cultured with the hydrogel extract **(bottom)** used for analysis for angiogenesis assay. Scale bars are 100 μm. **H.** Number of nodes detected from the angiogenesis assay, data presented as mean ± SD. n = 7, n=6, n=8, and n=5 for positive control, negative control, UBM-COL, and SKM-COL, respectively. P-values are calculated using Kruskal-Wallis non-parametric test with post-hoc analysis of Dunn's multiple comparisons, **P*<0.05, ****P*<0.001. **I.** Number of tubes detected from the angiogenesis assay, data presented as mean ± SD. n = 7, n=6, n=8, and n=8 for positive control, negative control, UBM-COL, and SKM-COL, respectively. P-values are calculated using Kruskal-Wallis non-parametric test with post-hoc analysis of Dunn's multiple comparisons, ***P*<0.005, ****P*<0.001. **J**. Length of detected tubes, data presented as mean ± SD. n = 7, n=6, n=8, and n=5 for positive control, negative control, UBM-COL, and SKM-COL, respectively. P-values are calculated using Kruskal-Wallis non-parametric test with post-hoc analysis of Dunn's multiple comparisons, ****P*<0.001.

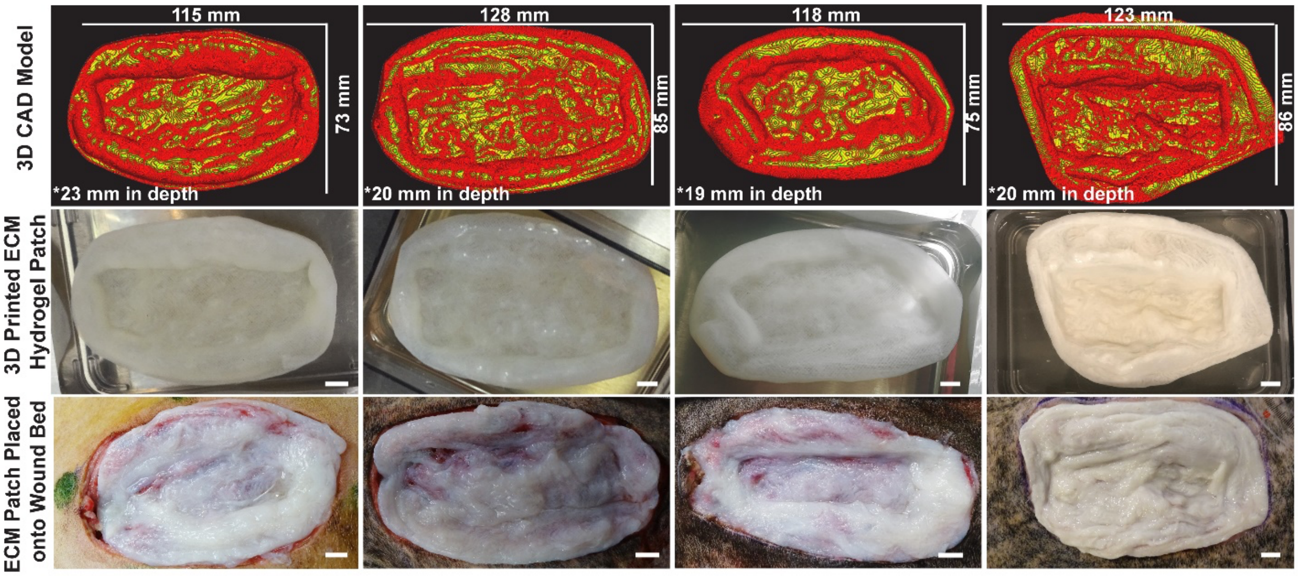

Supplemental Figure S3. Multiple Successful Implantations with Patches of Varying Sizes and Geometries. Slicing profiles (top row), photographs of bioprinted patches submerged in 50 mM HEPES (middle row), and photographs of implanted patches (bottom row) exemplify that the FRESH platform can successfully print patches with varying sizes and geometries. All scale bars are 1 cm.

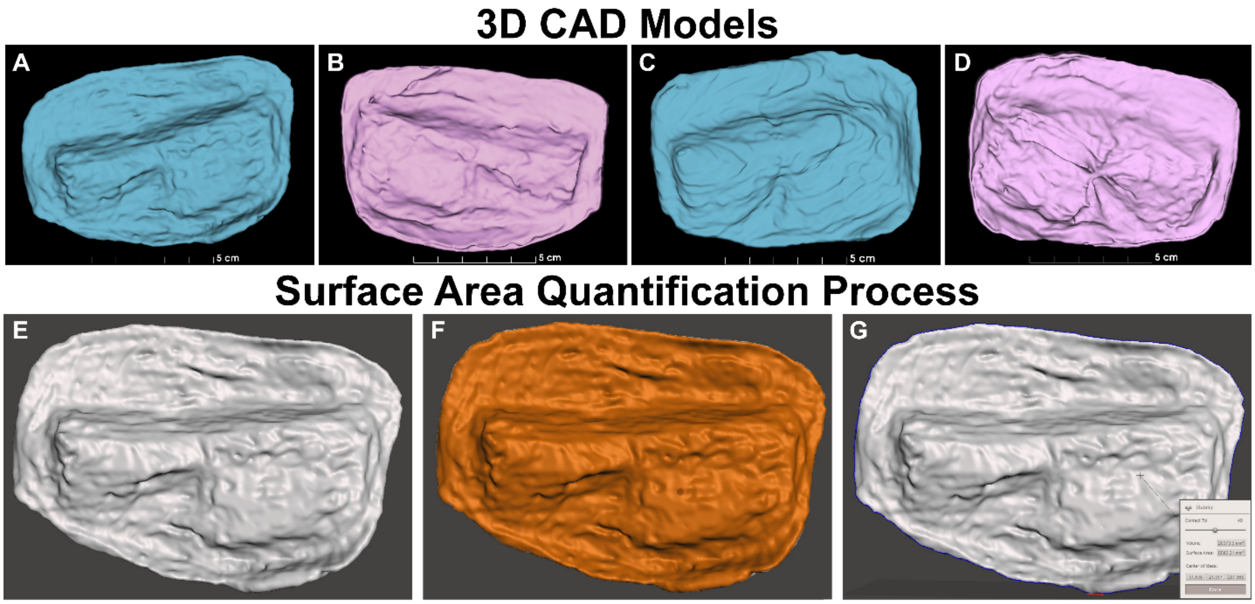

Supplemental Figure S4. Surface Area Quantification of 3D Models of dECM Prints and Wound Beds. Surface area comparison of the sides of the prints and wound that contact each other was used for conformality assessment. In order quantify the surface area, CAD models fabricated in 3D slicer of the dECM patch (**A**), the wound bed the patch was implanted on (**B**), the dECM flat sheet (**C**), and the wound bed the flat sheet was implanted on (**D**). Models were then imported into MeshMixer for surface area quantification. The surface area of the backside of the prints and the tops of the wound beds were quantified. The process of the surface area quantification of the implanted patch is shown as an example. (**E)** The STL file is imported into MeshMixer and the **(F)** “unwrap brush” under the selection window is used to highlight the backside of the patch. **(G)** The “separate” tool is then used to create two distinct surface (shown in the blue outline) and the “stability” tool under the analysis window provides the surface area.

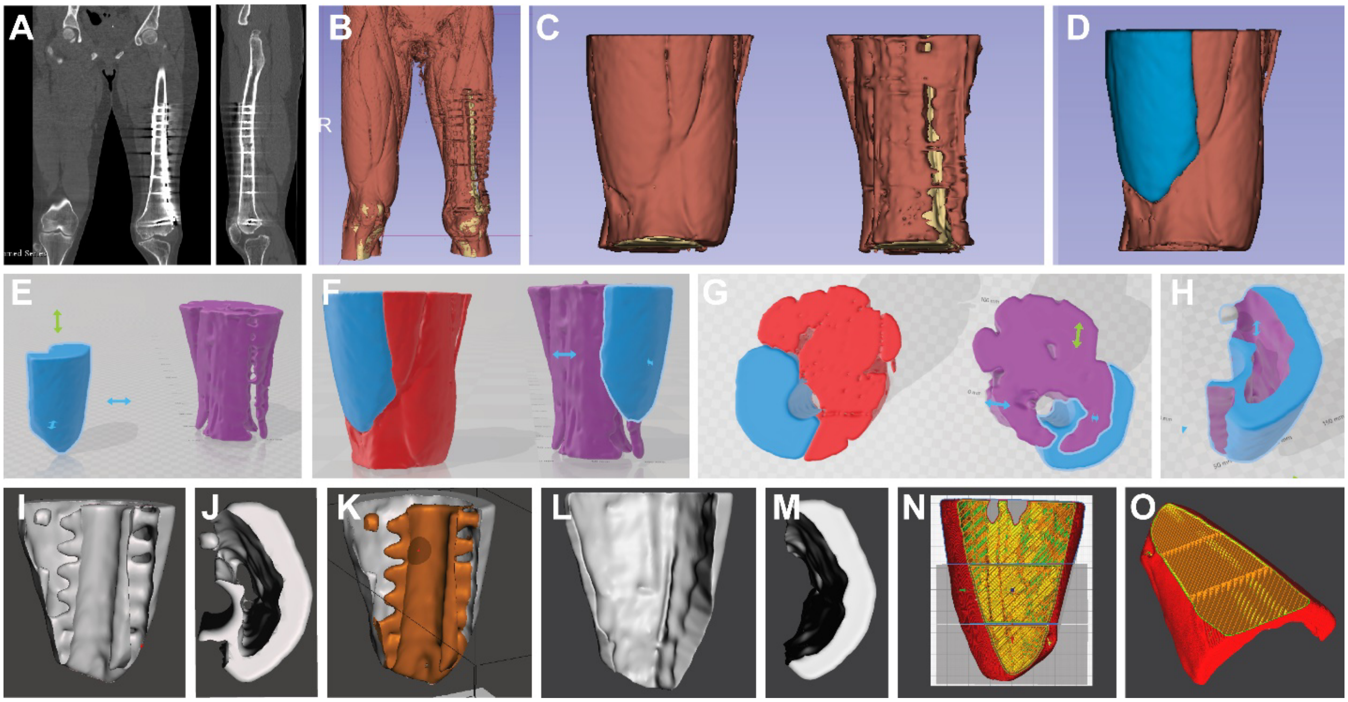

Supplemental Figure S5. Fabrication steps to create Human VML Scaffold. (A) CT scans of a human VML injury were provided. This was a complex injury that also included a femur fracture which required metal rods to help fix the bone. The metal in the injured leg made it hard in regions to section out the muscle due to excess noise. **(B)** We were able to segment a full model of the healthy leg, and a partial model of the injured leg (due to excess noise in the CT scans). **(C)** The models were cropped above the knee. **(D)** The healthy leg was used as the template for the scaffold. The vastus lateralis muscle was isolated in the healthy leg since a large amount of muscle mass was lost of the vastus lateralis on the injured leg. **(E)** The models were exported as STL files and imported into 3D builder. From here, the vastus lateralis muscle was "mirrored" to match the orientation of the injured leg. **(F)** The vastus lateralis muscle was overlayed over the injured leg. **(G)** Correct placement of the scaffold on the injured leg was verified by comparing alignment to the healthy leg. **(H)** A Boolean modifier (subtraction) was used to extract the difference between the overlayed vastus lateralis muscle and the injured leg. **(I-J)** The front **(I)** and top **(J)** view of the model imported into MeshMixer to remove the middle section and clean up. **(K)** The middle section (highlighted in orange) was cut from the model using the "Select – Edit – Discard" tool. **(L-M)** The model was smoothed in MeshMixer to remove any sharp regions and imported into Ultimaker Cura for G-code generation. Front **(L)** and top **(M)** views of the cleaned model. **(N)** Two rectangular modifiers were added to create three sections of the model. Infill percentage was then modified in each section to either 20%, 30%, or 40%. **(M)** Isometric view of the slicing path from the G-Code.
